# Paracrine Potential of the Human Adipose Tissue-Derived Stem Cells to Modulate Balance between Matrix Metalloproteinases and Their Inhibitors in the Osteoarthritic Cartilage In Vitro

**DOI:** 10.1155/2017/9542702

**Published:** 2017-07-27

**Authors:** Jaroslav Denkovskij, Edvardas Bagdonas, Ilona Kusleviciute, Zygmunt Mackiewicz, Ausra Unguryte, Narunas Porvaneckas, Sandrine Fleury, Algirdas Venalis, Christian Jorgensen, Eiva Bernotiene

**Affiliations:** ^1^Department of Regenerative Medicine, State Research Institute Centre for Innovative Medicine, Vilnius, Lithuania; ^2^Clinic of Rheumatology, Orthopaedics-Traumatology and Reconstructive Surgery, Vilnius University Faculty of Medicine, Vilnius, Lithuania; ^3^EFS Pyrénéés-Méditerranéé, Toulouse, France; ^4^INSERM U844, Hôpital Saint-Eloi and Hôpital Lapeyronie, Université Montpellier 1, Montpellier, France

## Abstract

Adipose tissue represents an abundant source of stem cells. Along with anti-inflammatory effects, ASC secrete various factors that may modulate metabolism of extracellular matrix in osteoarthritic (OA) cartilage, suggesting that the presence of ASC could be advantageous for OA cartilage due to the recovery of homeostasis between matrix metalloproteinases (MMPs) and their tissue inhibitors of metalloproteinases (TIMPs). To evaluate these effects, cartilage explants (CE) were cocultured with ASC for 3 and 7 days under stimulation with or without IL-1*β.* The pattern of gene expression in CE was modified by ASC, including the upregulation of *COL1A1* and *COL3A1* and the downregulation of *MMP13* and *COL10A1*. The production of MMP-1, MMP-3, and MMP-13 by ASC was not significant; moreover, cocultures with ASC reduced MMP-13 production in CE. In conclusion, active production of TIMP-1, TIMP-2, TIMP-3, IL-6, IL-8, and gelatinases MMP-2 and MMP-9 by ASC may be involved in the extracellular matrix remodelling, as indicated by the altered expression of collagens, the downregulated production of MMP-13, and the reduced chondrocyte apoptosis in the cocultured CE. These data suggest that ASC modulated homeostasis of MMPs/TIMPs in degenerated OA cartilage in vitro and might be favourable in case of the intra-articular application of ASC therapy for the treatment of OA.

## 1. Introduction

Osteoarthritis (OA) is a slowly progressing joint disease, where the rate of loss of collagens and proteoglycans of cartilage matrix exceeds the rate of deposition of newly synthesized molecules [1]. Catabolism of matrix proteins can be accomplished by several classes of enzymes; however, the metalloproteinases (MMPs) are generally considered predominantly responsible for connective tissue destruction in arthritic joints [2, 3]. The activity of MMPs is controlled through the activation of proenzymatic form and the inhibition of active enzymes by tissue inhibitors of metalloproteinases (TIMPs). The contribution to cartilage degradation in arthritis has been suggested for the excess of MMPs over TIMPs [4]. The increased expression of MMP-1, MMP-3, and MMP-13, which degrades structural collagens, including intact collagen type II, in osteoarthritic cartilage suggests a major role for these enzymes in cartilage degradation [2]. Gelatinases A and B (MMP-2 and MMP-9, resp.) digest the denatured collagens, gelatins, as well as some noncollagen matrix components of the joints.

Many studies imply the therapeutic potential of mesenchymal stem cells (MSC) for cartilage repair in OA. Adipose tissue-derived stem cells (ASC) are close to MSC from bone marrow in their anti-inflammatory and supportive for tissue repair effect [5]. The multipotent differentiation abilities [6] as well as accessibility, reproducibility, and ease of isolation of ASCs make them ideal candidates for musculoskeletal repair, and a number of ASC-based approaches for cartilage repair have progressed from preclinical animal studies into clinical trials [7]. There is growing evidence that regenerative properties of human ASCs could be explained by paracrine release of bioactive factors required to accelerate and direct tissue repair [8]. Recently, the anti-inflammatory effects of ASC were demonstrated in vitro and in vivo, and the intra-articular injections of ASC and MSC for the treatment of OA lesions have been tested in several clinical trials [9].

In the present study, we hypothesized that the paracrine chondroprotective effects of ASC on the OA cartilage would be advantageous due to the recovery of MMP/TIMP homeostasis. To avoid the dedifferentiation of chondrocytes, cultured in monolayer, or the synthesis of cartilage extracellular matrix (ECM) de novo in pellet cultures, we have chosen a model of human OA cartilage explants (CE), containing viable cells, readily surrounded by native OA cartilage ECM environment [10]. CE were cocultured or not with ASC, and the alterations in MMP and TIMP secretion by CE in response to ASC cocultures were analysed. The loss of cartilage in OA is not only a consequence of the enhanced degradation of ECM in response to catabolic factors but also a failure of cartilage repair once it begins to breakdown [11]. Therefore, we have chosen to also investigate the effects of ASC on the expression of a set of genes, reflecting metabolic state of chondrocytes in OA cartilage, including ECM components and markers of hypertrophy. Collagen type II, regulated by SOX9 transcription factor, and aggrecan were chosen as the essential markers of chondrogenesis [12]. Collagen type I is highly produced during fetal development or healing of cartilage, while in the healthy mature articular cartilage, it remains located on the surface. Collagen type III comprises more than 10% of total collagen in mature articular cartilage, and together with collagen type I, they both are reported to participate in matrix repair and/or remodelling [13, 14]. Hyaluronan and proteoglycan link protein 1 (*HAPLN1*) is a key component of the cartilage ECM that stabilizes aggregates of aggrecan and hyaluronic acid, while *COL10A1* and *MMP-13* were included as the genes related to chondrocyte hypertrophy [15].

Several types of culture conditions were investigated for coculture experiments in our study, including chemically defined serum-free medium, also referred to as incomplete chondrocyte medium (IM). For the better representation of therapeutic conditions, cell cultures were performed in incomplete chondrocyte medium (IM), additionally supplemented with human platelet growth factor-enriched plasma (PP), which is used for ASC expansion for clinical applications [16]. Moreover, to further reproduce the in vivo OA conditions and to evaluate the possible impact of inflammatory environment on the crosstalk between ASC and CE, the cocultures were stimulated with or without IL-1*β*, a principal elevated cytokine in OA [11, 17].

## 2. Materials and Methods

### 2.1. Coculture Experiments

**Figure 1:**
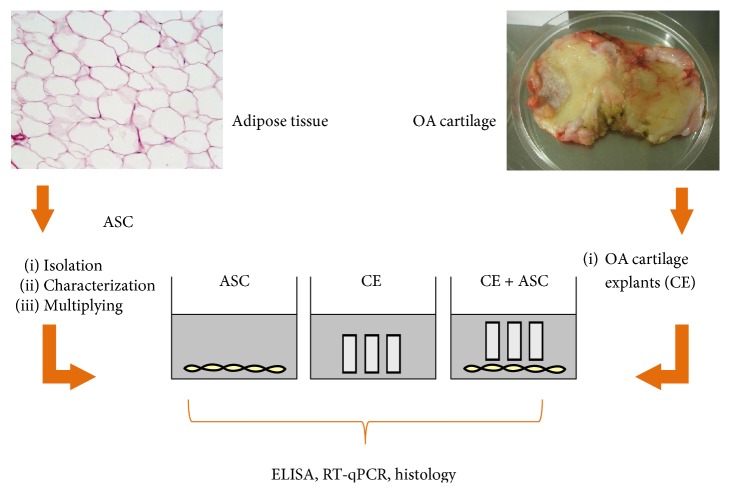
Scheme of coculture experiment of human osteoarthritic (OA) cartilage explants (CE) derived from cartilage remaining after joint replacement surgery, cultured alone or together with adipose tissue-derived stem cells (ASC) in monolayer. Experiments were performed in the following: (1) incomplete chondrocyte medium (IM), (2) IM supplemented with 2% human platelet growth factor-enriched plasma (PP), and (3) in IM + PP with interleukin- (IL-) 1*β*.

Samples of cartilage were obtained during the knee joint replacement surgery from patients with a grade IV OA, which according to the Kellgren and Lawrence five grades (0–IV), system is classified as severe and characterized by prominent osteophyte formation and articular surface erosion to the subchondral bone with gross geometric changes. The study protocol was approved by the Lithuanian Bioethics Committee at III “Respublikine Vilniaus universitetine ligonine”, and informed consents were obtained from the patients included into the study. Cartilage tissue was dissected from the locations with morphologically similar lesions. The removed pieces of the cartilage tissue were further chopped into the small explants of 1–3 mm on each side, mixed, and divided into the portions of 120 mg weight, which according to the published data [18] and the observed cell yields during our established enzymatic chondocyte isolation procedure, best correspond to ~5 × 10^5^ chondrocytes. Two days prior to cocultures, CE were preincubated in culture medium. Human ASC were isolated after enzymatic digestion of adipose tissue obtained from subcutaneous abdominal fat of healthy donors during liposuction procedure, and cell expansion of the stromal vascular fraction was performed using the procedures implemented for clinical applications (EFS, Toulouse, France) according to good manufacturing practice (GMP). All the isolated ASC products have been characterized as previously described [5, 19, 20] and corresponded to the following criteria: viability > 90%, positivity for CD90 and CD73 > 90%, CD105 > 80%, CD45 and CD14 < 2%, and CD34 < 10%, and no expression of hTert at the end of P0 [5, 20]. Triple differentiation potential also has been confirmed. All cell samples involved in this study were checked to be positive for the capacity of multipotent differentiation into adipogenic, osteogenic, and chondrogenic lineages [5]. For the cocultures, ASC were plated at 10^5^/well in a 6-well plate and cultured overnight for adherence. CE were directly placed on monolayer of ASC, while separate cultures of ASC or CE were used as controls (Figure 1). All cultures were performed on cartilage samples and ASC of 4 different allogeneic donors per experiment, resulting in 16 coculture combinations. Incomplete chondrocyte medium (IM) contained the following: DMEM with sodium pyruvate 4.5 g/L glucose (Biochrom), penicillin/streptomycin 100 U/mL (Biological Industries), proline 0.35 mM (Carl Roth), ascorbic acid phosphate 0.17 mM (Sigma Aldrich), supplemented or not with 2% human platelet growth factor-enriched plasma (PP), and heparin 1 U/mL. Platelet lysate has been prepared using GMP-grade protocol for large-scale expansion of human MSC from apheresis-derived clinical-grade platelet concentrates, as previously described [16, 20]. To further reproduce the OA conditions, cocultures were also stimulated with IL-1*β* 10 ng/mL (Gibco). On days 3 and 7 of cocultures, supernatants were collected, immediately frozen, and stored for later analysis, and CE were further processed for RT-qPCR and/or histology.

### 2.2. ELISA

Protein levels of TIMP-1, TIMP-2, MMP-1, MMP-3, MMP-13, MMP-2, MMP-9, IL-1*β*, TGF*β*3, TGF*β*2, thrombospondin-1 (R&D Systems), TIMP-3, VCAM-1 (Boster Biological Technology), TGF*β*1 (IBL International), MIA (Roche), IL-8 (Invitrogen), and aggrecan (DIAsource ImmunoAssays S.A.) in supernatants were measured on days 3 and 7 by commercially available ELISA kits according to the manufacturer's instructions. IL-6 was measured by flow cytometry using Diaclone DIAplex IL-6 kit (Gen-Probe), and glycosaminoglycans were measured by blyscan-sulphated glycosaminoglycan assay (Biocolor). ELISA data were normalized by subtracting the values of medium from the samples.

### 2.3. Total MMP Activity

Total MMP activity was determined in the supernatants on days 3 and 7, using SensoLyte 520 Generic MMP Assay Kit (Anaspec, USA), according to the manufacturer's protocol. The kit detects total activity of MMP-1, MMP-2, MMP-3, MMP-7, MMP-8, MMP-9, MMP-12, MMP-13, and MMP-14 based on a 5-FAM/QXL520 fluorescence resonance energy transfer (FRET) peptide, which is used as an MMP substrate. Supernatants were incubated 2 h with MMP substrate, and fluorescence signal was measured at Ex/Em = 490/520 nm (SpectraMax).

### 2.4. RNA Extraction from Cartilage Explants

At the end of coculture experiments (days 3 and 7), the cartilage explants (CE) were collected, flash-frozen in liquid nitrogen, and stored at −70°C. Frozen CE samples were homogenized with mortar and pestle, for 15 min precooled in liquid nitrogen. Cartilage powders were immediately suspended in 1 mL of QIAzol lysis buffer (Qiagen) and RNA extracted according to the manufacturer's protocol. Due to high content of copurified glycosaminoglycans, RNA was subsequently purified with RNeasy Mini Spin columns (Qiagen) according to the manufacturer's instructions. RNA concentration and purity were measured with the NanoPhotometer™ Pearl (Implen).

### 2.5. RT-qPCR

RNA samples were processed as previously described [21]. Briefly, they were treated with DNase I (Fermentas) and cDNA synthesis was performed with the Maxima® First Strand cDNA Synthesis Kit (Fermentas), according to the manufacturer's protocols. PCRs were performed using Maxima Probe qPCR Master Mix (2X) (Fermentas) and Stratagene MX-3005P detection instrument (Agilent Technologies). The TaqMan® Gene Expression Assays (Applied Biosystems) for 9 genes were used for gene expression analysis (Table 1). The PCR reaction volume was 25 *μ*L with 0.5 *μ*L of 20X Taqman® Gene Expression Assay mix. All reactions were run in triplicates. Cycle conditions were as follows: initial denaturation step for 10 min at 95°C, followed by 40 cycles of 15 s at 95°C for denaturation and 60 s for annealing and extension. Each RNA sample was controlled for genomic DNA contamination by reactions without reverse transcriptase (RT), and reagent contamination was checked by the reactions without template (NTC). Individual gene expression efficiencies were calculated with the LinRegPCR program [22]. Average gene expression efficiency for every single gene was calculated from the different runs and used for later relative gene expression quantification. The gene expression ratio (CE + ASC versus CE) was calculated using M.W. Pfaffl equation with the real reaction efficiencies [23]. For normalization of gene expression, the geometric mean of two reference genes—RPS9 and B2M, was used.

### 2.6. Histology

Zero to 14-point histological-histochemical grading system (HHGS) or Mankin score for the evaluation of excised and analyzed osteoarthritic cartilage samples was used. “0” point means no changes, while fully degraded cartilage—14 points. Summarised score of CE slightly varied in different depth and length of the samples from different patients. Analysis was performed using the portions of cartilage with structural compromise about 3 points, loss of metachromatic matrix staining—2 points, cellularity abnormality—1 point, and tidemark integrity violation—1 point. The integrated Mankin score of analyzed samples was 6–8 points.

For histochemical and immunohistochemical analysis, following 7 days cultivation in vitro, CE were fixed in 10% neutral formalin and embedded into paraffin. 3-micrometer sections were deparaffinised and processed for standard staining with toluidine blue (pH 2.0). Immunohistochemical staining with antibodies against active caspase-3, collagen type I, and collagen type X (Abcam) was performed after antigen retrieval with citrate buffer pH 6.0 at +98°C for 20 min and endogenous peroxidase blocking with 0.3% hydrogen peroxide for 15 min at room temperature. ABC staining kit (Santa Cruz) and 3.3-diaminobenzidine as a chromogen were used. Stained sections were evaluated and blindly scored independently by two histology experts.

Scoring system applied in histochemical and immunohistochemical evaluation: 0—no stained profiles; 1—few stained profiles/weak specific staining; 2—stained profiles occupy about 50% of analysed microscopic field/moderate specific staining; 3—stained profiles occupy about 75% of analysed microscopic field/strong specific staining; 4—stained profiles occupy 100% of analysed microscopic field/very strong specific staining.

### 2.7. Statistical Analysis

Data were analysed using SPSS 15.0 software and nonparametric Kruskal-Wallis or Mann–Whitney *U* test was used for comparisons. *p* values less than 0.05 were considered statistically significant.

## 3. Results

### 3.1. Effects of ASC on the Production of MMPs

**Figure 2:**
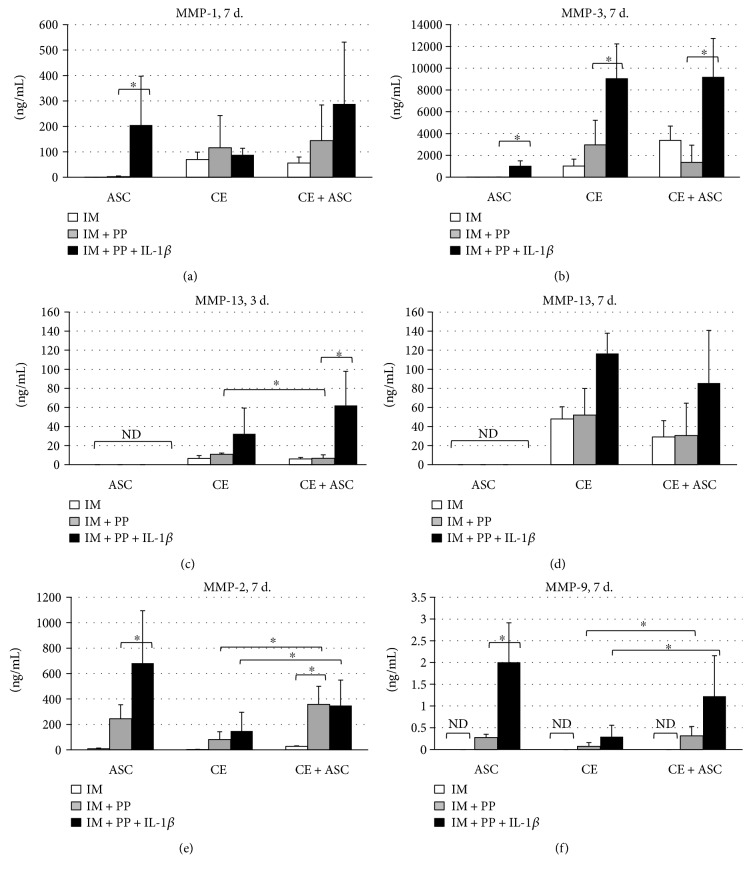
Secretion of matrix metalloproteinase- (MMP-) 1 (a), MMP-3 (b) on day 7, MMP-13 (c, d) on days 3 and 7, MMP-2 (e), and MMP-9 (f) on day 7 (in ng/mL), determined by ELISA in supernatants of cartilage explants (CE), ASC, and in cocultures (CE + ASC). IM—incomplete chondrocyte medium, IM + PP—IM supplemented with human platelet growth factor-enriched plasma (PP), and IL-1*β*—interleukin-1*β*. Data presented as mean ± standard deviation; *n* = 4–16; ^∗^*p* < 0.05; ND—not detected. The levels of MMPs obtained from day 3 experiments in the most of cases were similar to day 7; therefore, only day 7 demonstrated.

No production of MMP-13 was detected, while levels of MMP-1 and MMP-3 in supernatants of ASC cultured either in IM or IM + PP were marginal on day 3 and/or day 7 (Figures 2(a), 2(b), 2(c), and 2(d)). Relatively high levels of those MMPs were determined in the supernatants of CE, while no significant increase was detected in cocultures (CE + ASC) under the same two culture conditions (IM and IM + PP). Furthermore, the production of MMP-13 was significantly reduced when CE were cocultured with ASC for 3 days, and similar tendency was observed on day 7.

Following stimulation with IL-1*β*, significant increase in MMP-1 and MMP-3 production in ASC and MMP-3 in CE was determined. In cocultures, IL-1*β* stimulated the production of MMP-1, MMP-3, and MMP-13 but the levels were comparable to the ones produced by CE alone.

Gelatinases MMP-2 and MMP-9 were produced by both ASC and CE (Figures 2(e) and 2(f)). MMP-2 levels were significantly induced in ASC and CE + ASC by PP supplementation. IL-1*β* further strongly increased the secretion of MMP-2 in ASC alone but had no additional effect in CE + ASC. The production of MMP-9 in ASC, CE, and CE + ASC was very weak. Stimulation with IL-1*β* significantly increased the levels of MMP-9 in ASC and CE + ASC, while in the latter group to a lesser extent.

### 3.2. Production of TIMPs by ASC

**Figure 3:**
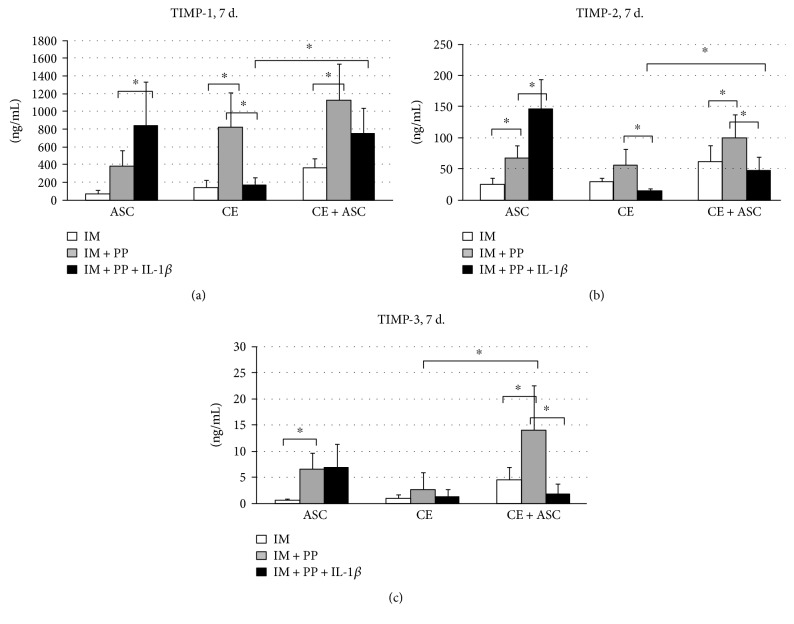
Secretion of tissue inhibitors of metalloproteinase- (TIMP-) 1 (a), TIMP-2 (b), and TIMP-3 (c), determined by ELISA in supernatants of cartilage explants (CE), adipose tissue-derived stem cells (ASC), and in cocultures (CE + ASC) on day 7. IM—incomplete chondrocyte medium, IM + PP—IM supplemented with human platelet growth factor-enriched plasma (PP), and IL-1*β*—interleukin-1*β*. Data presented as mean ± standard deviation; *n* = 4–16; ^∗^*p* < 0.05. The levels of TIMPs obtained from day 3 experiments in the most of cases were similar to day 7; therefore, only day 7 demonstrated.

Analysis of supernatants revealed the production of TIMP-1, TIMP-2, and TIMP-3 by both ASC and CE, which in all cases was augmented by PP (Figure 3). IL-1*β* had completely opposite effects on the production of TIMPs analysed, being stimulatory for the TIMP-1 and TIMP-2 in ASC and suppressive for the all measured TIMPs in CE.

Noteworthy, the levels of TIMPs in supernatants of CE + ASC at IM + PP conditions represent nearly combined amounts of corresponding TIMPs produced separately by CE and ASC, whereas in the presence of IL-1*β* (IM + PP + IL-1*β*), CE + ASC produced less TIMPs than each separately.

### 3.3. Secretion of Factors Related to Inflammation or Chondrogenesis

**Figure 4:**
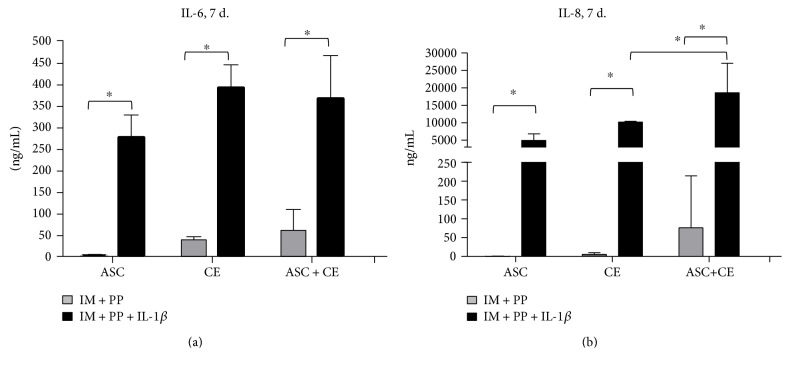
Secretion of IL-6 (a) and IL-8 (b) determined by ELISA in supernatants of cartilage explants (CE), adipose tissue-derived stem cells (ASC), and in cocultures (CE + ASC) on day 7. IM + PP—IM (incomplete chondrocyte medium) supplemented with human platelet growth factor-enriched plasma (PP), and IL-1*β*—interleukin-1*β*. Data presented as mean ± standard deviation; *n* = 4–16; ^∗^*p* < 0.05.

The levels of IL-6 produced by ASC in IM + PP were very low, as compared to CE; however, stimulation with IL-1*β* had a considerable effect on the production of IL-6 by both ASC and CE. Nevertheless, cocultures of CE with ASC did not significantly change the levels of IL-6. The lower production of IL-8 by ASC, as compared to CE was determined, which was further significantly elevated in CE + ASC. IL-1*β* stimulation resulted in a dramatic increase of IL-8 production in ASC, CE, and CE + ASC (Figure 4, Table 2).

The low production of soluble VCAM-1, a highly significant risk predictor of hip and knee joint replacement due to severe OA [24], was determined in supernatants of ASC (Table 2); however, it was abundantly secreted by CE.

ASC produced TSP-1, a factor known for its dual effects on articular cartilage, including antiangiogenic and antihypertrophic [25], in even somewhat higher quantities as compared to CE, however, cocultures resulted in no major effect on TSP-1 production.

The levels of melanoma inhibitory activity (MIA), a marker for chondrocyte differentiation [26], tended to be higher in supernatants of CE cocultured with ASC, as compared to CE alone on day 3, the effect being not observed on day 7. In addition, the production of ECM components was analyzed, and only traces of soluble glucosaminoglycans and no aggrecan secreted by ASC were determined, while those components were constitutively produced by CE. Cocultures with ASC or stimulation by IL-1*β* had no major effects on the levels of those soluble factors. Similarly, relatively low production of TGF-*β*1 was determined in ASC supernatants, as compared to CE, resulting in no significant changes in TGF-*β*1 levels in CE cocultured with ASC, under either culture conditions used (Table 2).

### 3.4. Effects of ASC on the Total MMP Activity of CE

**Figure 5:**
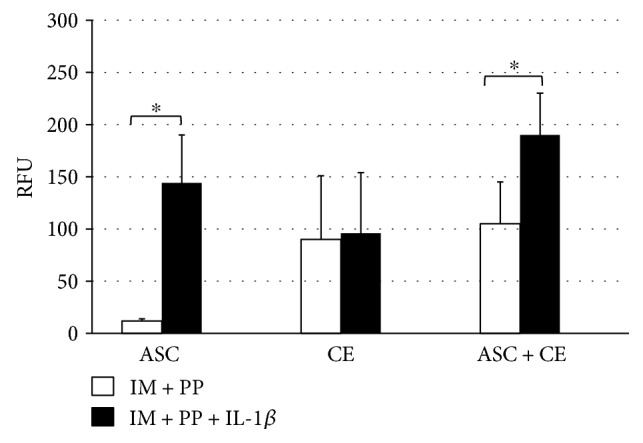
Total activity of MMPs (including MMP-1, MMP-2, MMP-3, MMP-7, MMP-8, MMP-9, MMP-12, MMP-13, and MMP-14) in supernatants of cartilage explants (CE), adipose tissue-derived stem cells (ASC), and in cocultures (CE + ASC) on day 7. After 2 h of incubation, fluorescence signal was measured at Ex/Em = 490/520 nm. IM + PP—IM supplemented with human platelet growth factor-enriched plasma (PP), IL-1*β*—interleukin-1*β*. Data presented as mean ± standard deviation, *n* = 4–16, ^∗^*p* < 0.05.

To evaluate a contribution of ASC to the enzymatic activity of the chondrocytes in CE, we have chosen the assay quantifying total MMP activity. Cocultures with ASC resulted in similar levels of active MMPs, as compared to CE alone, which were additionally augmented by IL-1*β* in ASC and cocultures but not in CE (Figure 5).

### 3.5. Changes in ECM-Related Gene Expression in Cocultured CE

**Figure 6:**
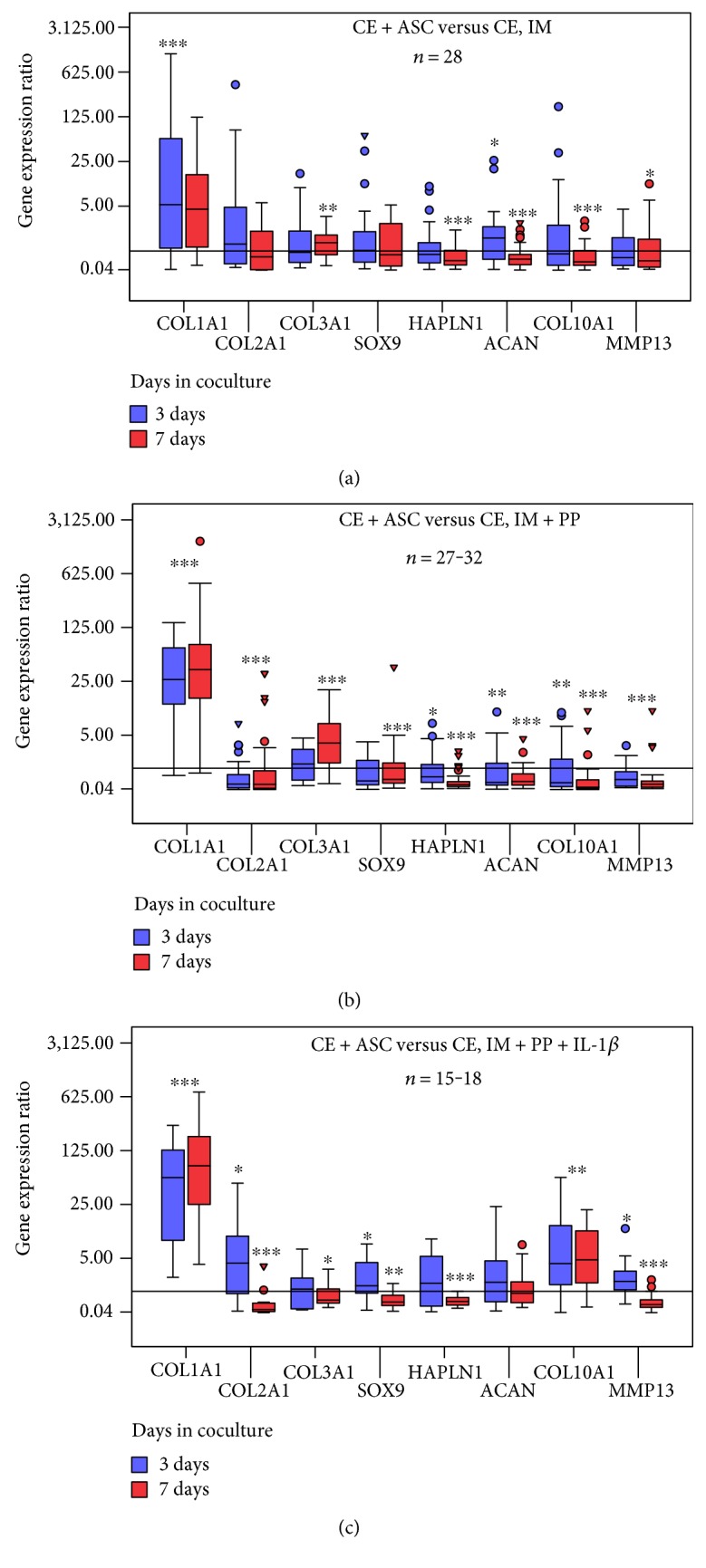
Distribution of the gene expression ratios in cartilage explants (CE) + adipose tissue-derived stem cells (ASC) versus CE in the following: (a) incomplete chondrocyte medium (IM) (*n* = 28), (b) in IM + human platelet growth factor-enriched plasma (PP) (*n* = 27/32), (c) in IM + PP+ IL-1*β*—interleukin-1*β* (*n* = 15/18). The box length represents the interquartile range with median. ▾—extreme cases with values more than 3 box lengths from the upper or lower edge of the box. ○—outliers with values between 1.5 and 3 box lengths from the upper or lower edge of the box. ^∗^*p* < 0.05; ^∗∗^*p* < 0.01; ^∗∗∗^*p* < 0.001.

The levels and pattern of gene expression in CE were obviously influenced by ASC and the culture medium used. When the coculture experiments were performed in incomplete chondrocyte medium (IM), there was a very high variation in gene expression after 3 days of cocultures (Figure 6(a)). Only the expression of *COL1A1* and *ACAN* genes significantly changed (upregulated 92.4- and 3.15-fold, resp.) in the presence of ASC. Seven days of CE cocultures with ASC in IM resulted in significantly higher expression of *COL1A1* and *COL3A1* genes (*p* < 0.01), unaltered *SOX9* and *COL2A1,* while significantly reduced (*p* < 0.05) expression of the rest of the analyzed genes was observed.

Similar gene expression pattern with the less variation and thus more pronounced changes was observed in CE, cocultured with ASC in the medium with PP (Figure 6(b)). After 3 days of coculture, significant 32-fold upregulation of *COL1A1* expression (*p* < 0.001) and downregulation of *COL2A1*, *COL10A1*, *HAPLN1*, *ACAN*, and *MMP13* (*p* < 0.05) by ASC were determined.

More pronounced changes were observed on day 7: *COL1A1* and *COL3A1* were significantly upregulated by 102- and 3-fold, respectively, (*p* < 0.001), while the rest of the genes analyzed were significantly (*p* < 0.001) downregulated.

Cocultures of CE with ASC in medium containing IL-1*β* (IM + PP + IL-1*β*) resulted in a significant (*p* < 0.001) upregulation of *COL1A1*, *COL2A1*, *COL10A1*, *SOX9*, and *MMP13* genes (58-, 6.35-, 7.84-, 1.76-, and 1.85-fold, resp.) in CE on day 3 (Figure 6(c)). Seven days of coculturing under the same conditions showed upregulation of only *COL1A1* and *COL10A1* genes (124- and 4.2-fold, *p* < 0.01), while *COL2A1*, *SOX9*, *HAPLN1*, and *MMP13* were significantly (*p* < 0.01) downregulated.

Summarizing the results of all cocultures, *COL1A1* gene appeared to be upregulated in CE by ASC in all cases independently of culture medium constitution. Genes related to hyperthrophy, *COL10A1* and *MMP13*, as well as chondrogenic markers, including *COL2A1, ACAN, HAPLN1*, and *SOX9*, tended to be progressively downregulated by ASC with the longer duration of coculture.

### 3.6. Histological and Immunohistochemical Examination of Cocultured CE

**Figure 7:**
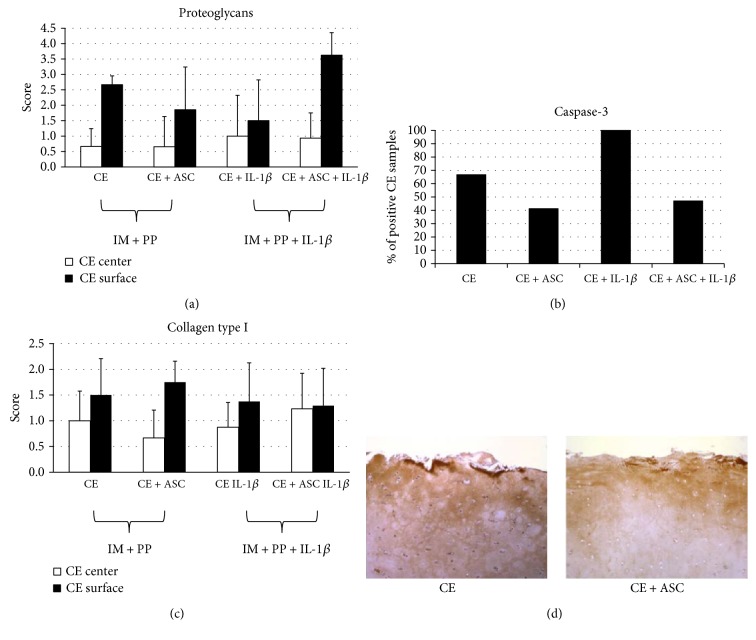
Histochemical evaluation of extracellular matrix production and immunohistochemical evaluation of apoptosis in cultured human osteoarthritic cartilage explants (CE, *n* = 4) cultured alone or cocultured with adipose tissue-derived stem cells (ASC, *n* = 4) in IM + PP, stimulated or not with interleukin- (IL-) 1*β* on days 3 and 7, *n* = 4–17. (a) Proteoglycans, safranin O staining; (b) active caspase-3, immunostaining of CE; (c) collagen type I, immunostaining of CE; (d) representative sample of immunohistochemical staining with antibodies against collagen type I (brown) of human osteoarthritic CE for 7 days; 400x magnification. Scoring system: 0—no stained profiles; 1—few stained profiles/weak specific staining; 2—stained profiles occupy about 50% of analysed microscopic field/moderate specific staining; 3—stained profiles occupy about 75% of analysed microscopic field/strong specific staining; and 4—stained profiles occupy 100% of analysed microscopic field/very strong specific staining. Data in Figure 2(b) is expressed as % of samples positive for caspase-3.

Histological and immunohistochemical examination showed a weak tendency of improvement in ECM content and cell status in CE, cocultured with ASC, as compared to CE alone in medium with PP. A tendency to a higher toluidine blue staining intensity was observed in CE + ASC, as compared to CE alone (Figure 7(a)). Low expression of active caspase-3 was observed in all layers of the osteoarthritic cartilage, in both CE and CE + ASC groups, on days 3 and 7. Caspase-3-positive CE samples were less frequent in the presence of ASC, both under IM + PP and IM + PP + IL-1*β* conditions (Figure 7(b)). Most abundant accumulation of collagen type I protein was observed at the native surface of the cartilage, but it was also present in the territorial zone surrounding chondrons, as well as in the interterritory area (Figure 7(c) and 7(d)). There was a high interpatient variability in deposition of collagen type I in cartilage samples, complicating detection of effects of ASC or IL-1*β*. (Figure 7(d)). Collagen type X was weakly expressed in all CE samples, and effects of cocultures were not evident (data not shown). Noteworthy, no cell growth was observed on the surface of CE, suggesting that the contamination of CE with ASC is unlikely.

## 4. Discussion

In the present study, we investigated the interactions between ASC and OA chondrocytes, remaining in their natural environment of ECM, namely CE, in cocultures in vitro.

MMP-1, MMP-3, and MMP-13 are involved in the direct degradation of intact structural collagens, including collagen type II [2]. Our results demonstrate the absence of the substantial production of those MMPs by ASC in vitro, implying safety of those cells for OA cartilage. Moreover, a significant ASC-mediated downregulation of MMP-13 gene expression and a decrease in protein level were determined in CE. MMP-13 plays a predominant role in the early stages of OA, suggesting that its inhibition might prevent escalation of the disease and mediate antihypertrophic effects [3, 25]. Therefore, the downregulation of MMP-13, observed in our study, suggests a protective role of ASC for the cartilage and implies beneficial effects of potential ASC therapy in OA.

MMP-2 and MMP-9 are generally considered as factors responsible for the enhanced ECM degradation in the cartilage [27]. However, their natural substrates are collagens, denatured by other MMPs, including MMP-1, MMP-3, and MMP-13 [28], suggesting that gelatinases MMP-2 and MMP-9 are not harmful to an intact ECM structure of cartilage but rather involved in the clearance of the products of collagen metabolism. This may also explain the elevated levels of gelatinases in synovial fluid observed during OA [29]. The role of these enzymes seems controversial in cartilage, whereas they are likely to have favourable effects in the pathogenesis of arthritis, including activated cartilage ECM remodelling and possible immunosuppressive activities [29, 30]. In the present study, considerably increased levels of the gelatinases in supernatants of cocultures, as compared to those of CE alone, suggest fostered elimination of the denatured collagens from cartilage if ASC therapy for OA treatment was used. These data are also in agreement with the previously reported production of MMP-2 and MMP-9 by MSC and ASC, which was shown to be associated with their migratory activity [31]. Noteworthy, out of all the MMPs tested, only the production of MMP-2 was upregulated by PP.

Another important finding of the present study is that ASC actively produce TIMP-1, TIMP-2, and TIMP-3, which control the activity of ECM-degrading enzymes. Noteworthy, ASC produced similar amounts of TIMPs, to those secreted by CE, and they were additionally augmented by PP in both cases. However, under IL-1*β* stimulation, the production of TIMPs was greatly increased only in ASC, while in CE, on the contrary, they became lower. To the best of our knowledge, these results, for the first time, demonstrate the opposite response of TIMP production by the chondrocytes and mesenchymal cells to the inflammatory stimuli. Overall, these results demonstrate the obvious role of milieu, namely, of rich in growth and/or inflammatory environment factors on the crosstalk between CE and ASC. Importantly, secretory profile of ASC seems not harmful for the cartilage but rather chondroprotective under either culture conditions tested.

No or low production of GAGs, aggrecan, and MIA was observed in supernatants of ASC, neither their secretion was enhanced in cocultures, suggesting that stem cells are unlikely to directly contribute to ECM synthesis in cartilage.

The results of the MMP total activity assay suggest increased general enzymatic activity in CE supernatants in the presence of ASC. The difference of the substrate of the assay (commercially undisclosed, FRET peptide) from the composition of a native cartilage should be taken into consideration, when extrapolating the results of this assay to the possible effects on the intact covalently bound collagen structures in cartilage in vivo. Nevertheless, the results of this assay further imply active participation of ASC in the metabolism of collagen components and cartilage remodelling. As gelatinases were the only MMPs, produced at considerable levels by ASC, they are likely to contribute to the high total activity in supernatants of the cells.

We next analysed gene expression of the key components of hyaline cartilage, including collagens type I, II, and III, aggrecan, SOX9, and link protein, seeking to determine if the activity of ECM production was altered in CE during cocultures with ASC. However, no upregulation of those genes by ASC, with the exception of *COL1A1* and *COL3A1*, was observed. Unaltered or downregulated gene expression of collagen type II, in some cases associated with the decreased aggrecan expression, has also been previously reported in chondrocytes in monolayer, cocultured with ASC [5] and MSC [3], or in CE under MSC-conditioned medium [32].

Dramatical upregulation of *COL1A1* gene expression in CE already on day 3 and additional increase on day 7 of cocultures with ASC, observed in our study, implies stimulated initial stage of OA cartilage healing. The upregulation of *COL1A1*, induced by ASC, to the best of our knowledge, has never been previously reported; moreover, it was not affected by the coculture medium composition or stimulation with IL-1*β*. Collagen type I is generally considered as an indicator of fibrosis or dedifferentiation of chondrocytes in monolayer, which is not desirable in hyaline cartilage construction [33]. However, elevated expression of collagen type I in immature articular cartilage and additional increase under stimulation with growth factors has been previously demonstrated [13]. Furthermore, it is constitutively located on the surface of mature articular cartilage [34], implying essential role for collagen type I in hyaline cartilage composition. In the study on human OA cartilage regeneration using autologous chondrocyte implants, more than 53 times increased collagen type I protein content was found in all layers of healing hyaline cartilage at the initial stage [35]. Therefore, we hypothesize that the observed increased expression of *COL1A1* in CE, cocultured with ASC, might represent an initial attempt to regenerate the damaged surface of OA cartilage, which possibly later will be replaced by the synthesis of collagen type II. Furthermore, we detected the suppressed *COL1A1* expression in CE by IL-1*β* (data not shown), a factor well known for its inhibitory effects on chondrogenesis [36]. Noteworthy, an increase in collagen type I expression has been previously reported when chondrocytes where cultured in 3D but not 2D environment [3]. An upregulation of *COL3A1* gene expression in cocultured explants under IM and IM + PP conditions was also observed in our study on day 7. Collagen type III was shown to be covalently linked to the surface of type I collagen fibrils in many tissues [37], while in articular cartilage, it is extensively cross-linked to the surface of type II collagen fibrils, suggesting a role of collagen type III in matrix reinforcement and a healing response to tissue damage. It is in agreement with the studies of Wu et al. showing that type III collagen is synthesized as a modifier of existing fibril networks in response to tissue and matrix damage [38]. Therefore, stimulation of collagen type III synthesis in the cartilage explants by ASC further implies contribution of ASC to cartilage reparation.

We also observed reduced expression of the genes responsible for hypertrophy and cartilage ECM degradation (*COL10A1* and *MMP13*) in CE cocultured with ASC. The downregulation of *COL10A1*, and particularly *MMP-13*, both at gene and protein levels, implies strong antihypertrophic and chondroprotective effects of ASC.

In the present study, IL-1*β* highly stimulated production of IL-6 and IL-8 by both ASC and CE. IL-6 and IL-8 are generally considered as proinflammatory cytokines; however, although they are produced by MSC and ASC, the downregulation of the inflammatory responses of those cells has been repeatedly reported [36]. Furthermore, both the pro- and anti-inflammatory roles of IL-6 have been reported, depending on the experimental model used and the activated signalling mode in responder cells [39].

Histological examination revealed trends of beneficial cartilage-preserving activities of ASC on CE, including improved ECM content and most importantly, less expressed apoptosis in cocultures. Significance of chondrocyte apoptosis in pathogenesis of OA has been demonstrated decades ago, and therapeutic value of its inhibition has been suggested [40]. These results imply that inhibition of apoptosis might appear as one of the potential mechanisms of favourable effects during ASC therapy for OA.

As the significant increase of *COL1A1* gene expression was determined in cocultured CE, we were seeking to investigate those effects at the protein level. Similarly to the previous reports [14], we observed that collagen type I is naturally most abundantly located at the surface of the cartilage, whereas the effects of ASC or IL-1*β* on its deposition were not obvious. We speculate that day 7 might still be a short period for the demonstration of cartilage response to ASC histologically. Similarly to van Buul et al. [32], where 2 days cocultures were performed, we hypothesize that days 3 and 7 are also indicative of an early response, whereas investigation of later events taking place in cocultures would further contribute to the elucidation of a crosstalk between ASC and CE, including ECM production turnover.

The stimulated anabolism of ECM, as indicated by the increased expression of *COL1A1* and to a lesser extent *COL3A1* in cocultured CE suggests an early phase of enhanced cartilage reparation. The production of MMP-2 and MMP-9, resulting in elevated enzymatic activity in cocultures, is likely to be implicated. We speculate that the gelatinase-mediated increased elimination of collagens, degraded by MMP-1 and MMP-13, may signal to induce the anabolic effects, thus, initiating reparative processes in cartilage. In addition, the production of TIMPs and the inhibition of MMP-13 by ASC may counteract an increased cartilage degradation observed during OA. Moreover, those beneficial properties of ASC were demonstrated in the presence of IL-1*β*, emphasizing suitability of their application under inflammatory conditions, as in OA. Generally, the trends of ASC effects were similar on both days 3 and 7, suggesting a fast response of CE to ASC in cocultures.

## 5. Conclusions

Taken together, the results of the present study suggest a protective role for ASC on OA cartilage by modulating the activity of cartilage-degrading enzymes and thus preventing ECM from degradation or even stimulating its reparation. The production of TIMPs and gelatinases by ASC may be involved in cartilage remodelling, as indicated by the increased *COL1A1* and *COL3A1* expression, the downregulated production of MMP-13, and the reduced chondrocyte apoptosis in the cocultured CE. These data suggest that ASC modulated reparation of degenerated OA cartilage in vitro. These effects seem safe and might be favourable in case of the intra-articular application of ASC therapy for the treatment of OA.

## Figures and Tables

**Table 1:** The TaqMan Gene Expression Assays used for gene expression analysis in CE.

Gene, assay ID	Encoded protein
*RPS9* Hs02339424_mL	40S ribosomal protein S9
*B2M* Hs00984230_mL	Beta-2 microglobulin
*HAPLN1* Hs01091997_mL	Hyaluronan and proteoglycan link protein 1
*COL1A1* Hs00164004_mL	Collagen type I, alpha 1
*COL2A1* Hs01060345_mL	Collagen type II, alpha 1
*COL3A1* Hs00943809_mL	Collagen type III, alpha 1
*COL10A1* Hs00166657_mL	Collagen type X, alpha 1
*ACAN* Hs00153936_mL	Aggrecan
*MMP13* Hs00233992_mL	Matrix metalloproteinase-13
*SOX9* Hs00165814_mL	Transcription factor SOX-9

**Table 2:** The levels of secreted factors related to inflammation and chondrogenesis.

Secreted factor, coculture duration, and medium conditions	Concentration, ng/mL		
	ASC	CE	ASC + CE
VCAM-1			
3 d. IM + PP	0.0	20.9 ± 8.4	25.7 ± 13.8
3 d. IM + PP + IL-1*β*	2.1 ± 1.5	83.3 ± 49.8	32.6 ± 13.4
7 d. IM + PP	7.3 ± 12.7	43.5 ± 27.6	39.6 ± 28.7
7 d. IM + PP + IL-1*β*	4.2 ± 4.9	19.1 ± 7.1	13.8 ± 11.6
TSP-1			
7 d. IM + PP	2519.1 ± 1164.4	2031.1 ± 1599.5	1729.5 ± 769.6
7 d. IM + PP + IL-1*β*	2076.0 ± 1565.0	1226.1 ± 239.0	1697.4 ± 783.4
MIA			
3 d. IM + PP	0.1 ± 0.0	14.4 ± 13.1	28.6 ± 19.5
3 d. IM + PP + IL-1*β*	0.1 ± 0.2	6.2 ± 6.9	18.4 ± 14.1
7 d. IM + PP	0.0 ± 0.0	35.6 ± 12.6	24.0 ± 18.44
7 d. IM + PP + IL-1*β*	0.0 ± 0.0	16.9 ± 9.3	14.4 ± 13.4
Aggrecan			
3 d. IM + PP	0.0	4.0^∗^10^5^ ± 2.3^∗^10^5^	4.1^∗^10^5^ ± 1.4^∗^10^5^
3 d. IM + PP + IL-1*β*	0.0	3.8^∗^10^5^ ± 1.1^∗^10^5^	4.4^∗^10^5^ ± 2.2^∗^10^5^
7 d. IM + PP	0.0	5.0^∗^10^5^ ± 1.6^∗^10^5^	5.6^∗^10^5^ ± 1.9^∗^10^5^
7 d. IM + PP + IL-1*β*	0.0	3.5^∗^10^5^ ± 6.9^∗^10^5^	4.5^∗^10^5^ ± 1.8^∗^10^5^
Glycosaminoglycan			
7 d. IM + PP	1.1 ± 0.5	93.9 ± 26.1	96.4 ± 38.1
7 d. IM + PP + IL-1*β*	1.5 ± 0.7	114.7 ± 22.3	97.8 ± 24.1
TGF *β*1			
3 d. IM + PP	2120.2 ± 1106.9	17753.3 ± 2060.0	15901.3 ± 7041.7
3 d. IM + PP + IL-1*β*	2995.9 ± 527.8	14087.7 ± 944.2	17175.4 ± 2281.5
7 d. IM + PP	2965.7 ± 481.4	19203.6 ± 933.7	18099.8 ± 5582.5
7 d. IM + PP + IL-1*β*	2694.8 ± 816.8	17122.7 ± 2230.1	14212.5 ± 6798.3

Table 2 represents the levels of vascular cell adhesion molecule 1 (VCAM-1), thrombospondin-1 (TSP-1), melanoma inhibitory activity (MIA), aggrecan, glycosaminoglycan, and transforming growth factor (TGF) *β*1, measured by ELISA in supernatants of cartilage explants (CE), adipose tissue-derived stem cells (ASC), and cocultures (CE + ASC). Incomplete chondrogenic medium supplemented with human platelet growth factor-enriched plasma—IM + PP, and supplemented with IL-1*β*—IM + PP + IL-1*β*. ^∗^*p* < 0.05, when compared to ASC + CE versus CE.

## Abbreviations

*ACAN:*: Aggrecan
ASC: Adipose tissue-derived stem cells
B2M: Beta-2 microglobulin
CE: Cartilage explant
*COL10A1*: Collagen type X, alpha 1
*COL1A1*: Collagen type I, alpha 1
*COL2A1*: Collagen type II, alpha 1
ECM: Extracellular matrix
*HAPLN1*: Hyaluronan and proteoglycan link protein 1
IL-: Interleukins
IM: Incomplete chondrocyte medium
MIA: Melanoma inhibitory activity
MMP-: Matrix metalloproteinase- (1, 2, 3, 9, and 13)
MSC: Mesenchymal stem cells
PP: Platelet growth factor-enriched plasma
*RPS9*: 40S ribosomal protein S9
*SOX9*: Transcription factor SOX-9
TGF *β*1: Transforming growth factor *β*1
TSP-1: Thrombospondin-1
TIMP-: Tissue inhibitors of metalloproteinase- (1, 2, and 3)
2D: Two-dimensional space
3D: Three-dimensional space
VCAM-1: Vascular cell adhesion molecule 1.
